# Epidemiology of geographic disparities in heart failure among US older adults: a Medicare-based analysis

**DOI:** 10.1186/s12889-022-13639-2

**Published:** 2022-07-01

**Authors:** Bin Yu, Igor Akushevich, Arseniy P. Yashkin, Anatoliy I. Yashin, H. Kim Lyerly, Julia Kravchenko

**Affiliations:** 1 Department of Surgery, Duke University School of Medicine, Durham, NC 27710 USA; 2 Social Science Research Institute, Duke University, Durham, NC 27710 USA; 3 Department of Epidemiology and Health Statistics, School of Public Health, Wuhan University, Wuhan, 430071 China

**Keywords:** Heart failure, Geographic disparities, Time trend, Mortality, Incidence-based mortality, Incidence, Prevalence, Survival

## Abstract

**Background:**

There are prominent geographic disparities in the life expectancy (LE) of older US adults between the states with the highest (leading states) and lowest (lagging states) LE and their causes remain poorly understood. Heart failure (HF) has been proposed as a major contributor to these disparities. This study aims to investigate geographic disparities in HF outcomes between the leading and lagging states.

**Methods:**

The study was a secondary data analysis of HF outcomes in older US adults aged 65+, using Center for Disease Control and Prevention sponsored Wide-Ranging Online Data for Epidemiologic Research (CDC WONDER) database and a nationally representative 5% sample of Medicare beneficiaries over 2000–2017. Empiric estimates of death certificate-based mortality from HF as underlying cause of death (CBM-UCD)/multiple cause of death (CBM-MCD); HF incidence-based mortality (IBM); HF incidence, prevalence, and survival were compared between the leading and lagging states. Cox regression was used to investigate the effect of residence in the lagging states on HF incidence and survival.

**Results:**

Between 2000 and 2017, HF mortality rates (per 100,000) were higher in the lagging states (CBM-UCD: 188.5–248.6; CBM-MCD: 749.4–965.9; IBM: 2656.0–2978.4) than that in the leading states (CBM-UCD: 79.4–95.6; CBM-MCD: 441.4–574.1; IBM: 1839.5–2138.1). Compared to their leading counterparts, lagging states had higher HF incidence (2.9–3.9% vs. 2.2–2.9%), prevalence (15.6–17.2% vs. 11.3–13.0%), and pre-existing prevalence at age 65 (5.3–7.3% vs. 2.8–4.1%). The most recent rates of one- (77.1% vs. 80.4%), three- (59.0% vs. 60.7%) and five-year (45.8% vs. 49.8%) survival were lower in the lagging states. A greater risk of HF incidence (Adjusted Hazards Ratio, AHR [95%CI]: 1.29 [1.29–1.30]) and death after HF diagnosis (AHR: 1.12 [1.11–1.13]) was observed for populations in the lagging states. The study also observed recent increases in CBMs and HF incidence, and declines in HF prevalence, prevalence at age 65 and survival with a decade-long plateau stage in IBM in both leading and lagging states.

**Conclusion:**

There are substantial geographic disparities in HF mortality, incidence, prevalence, and survival across the U.S.: HF incidence, prevalence at age 65 (age of Medicare enrollment), and survival of patients with HF contributed most to these disparities. The geographic disparities and the recent increase in incidence and decline in survival underscore the importance of HF prevention strategies.

**Supplementary Information:**

The online version contains supplementary material available at 10.1186/s12889-022-13639-2.

## Introduction

Prominent geographic disparities in life expectancy (LE) among older adults are present in the United States (US) with the highest 2017 LE observed in Hawaii and the lowest in Mississippi [1]. The etiologies underlying these disparities are complex, and potential causes may include human biology and genetic risk, behavioral, mental health and socio-environmental factors, as well as variations in access to health care and healthcare utilization. Nonetheless, the reasons for the disparities between the states with highest (leading states) and lowest (lagging states) LE are not fully understood. Understanding how disease-specific mortality contributes to geographic disparities in LE is important for optimization of health policy and interventions aimed at mitigating the LE gap.

As the leading contributor (explaining approximately 40% of the total differences in LE) to geographic disparities in LE in the US [2], heart failure (HF) accounted for approximately one in eight deaths in the U.S. in 2017 [3]. About 6.2 million adults were living with HF in 2013–2016 [4], and a projected 71% of all HF cases will be among adults aged 65+ in 2030 [5]. While sex and racial disparities in HF risks and mortality are well studied [6, 7], the substantial geographic disparities in HF mortality [8–10] received less attention. This presents a potential future problem as the prevalence of HF driven primarily by population aging [11] is expected to increase substantially within the next decades and will likely surpass the prevalence of other cardiovascular diseases [12]. Furthermore, the gradual decline of HF mortality observed over the past few decades [10, 13] has been reversing since 2012 [9, 10].

In this study, we investigate several scenarios to explain the variations of HF mortality across the U.S. We hypothesize that regions with higher HF-specific and total mortality have: (a) a higher HF incidence; (b) poorer survival of patients with HF; (c) higher pre-existing prevalence of HF at the time of entring (age 65) the Medicare – the primary payer for health service in older U.S. adults.

## Methods

### Data

Two sources of data were used in this study, both spanning the 2000–2017 period. Data on HF mortality in populations aged 65+ were extracted from the Wide-Ranging Online Data for Epidemiologic Research (WONDER) at the U.S. Centers for Disease Control and Prevention (CDC) which used the International Classification of Disease (ICD) code I50 (10th Revision) to ascertain a HF diagnosis during the study period [14]. Data on HF incidence-based mortality (IBM), incidence, prevalence, and survival were extracted from the 5% sample of over 5 million U.S. Medicare beneficiaries (Part A and Part B) [15]. A patient was excluded if 20% or more of his/her months were without Medicare coverage. The ICD codes 428 (9th Revision) and I50 (10th Revision) were used to ascertain a HF diagnosis.

To quantify geographic disparities in HF outcomes, all the U.S. states were ranked based on the age-standardized all-cause mortality in the 2015 age 65+ population [16], and the eight states with the lowest mortality were categorized as leading (Hawaii, Florida, Arizona, Connecticut, Minnesota, and Colorado, California, and New York), while the eight states with the highest mortality were categorized as lagging (Arkansas, Tennessee, Louisiana, Oklahoma, Kentucky, Alabama, Mississippi, and West Virginia). The states of California and New York were not included in the main analysis due to their higher percentage of urbanized areas and much higher population counts than in other states from the leading and especially from the lagging group. We tested the effect of this assumption in a sensitivity study (Supplemental eFigure 1).

### Variable measures

Annual death certificate-based mortality (CBM) (2000–2017) from HF as the underlying cause of death (CBM-UCD) and multiple causes of death (CBM-MCD) were drawn from the CDC WONDER database [14]. CBM-UCD was computed based on the number of deaths caused by HF and the total population in a specific year; CBM-MCD was computed using the number of deaths from any cause as long as HF was listed as a comorbidity in a specific year.

Annual IBM, HF incidence, and prevalence were computed based on the number of events and the total person-year in a specific year based on the Medicare database. IBM refers to the all-cause mortality with a priori diagnosis of HF; it was computed as the number of all-cause deaths in individuals with HF. Age at HF onset was identified using a previously published algorithm [17]. Detailed calculation of year-specific measures identified from Medicare trajectories can be found in the Supplemental Methods. One-, three- and five-year survival rates were calculated based on the date of death available in the Medicare records.

### Statistical analysis

The characteristics of the study sample were presented in Supplemental eTable 1. The age-standardized rates of CBMs, IBM, incidence, prevalence, prevalence at age 65, and survival after HF diagnosis (1-year, 3-year, and 5-year) were calculated based on the US 2000 standard population. The temporal trends of HF outcomes were plotted for (1) the leading and lagging states, (2) sex-specific patterns, and (3) race-specific patterns. The point estimates and 95% confidence intervals (CIs) were computed. To avoid the overplotting, the 95% CIs were not plotted in the figures of the temporal trends.

The Cox proportional hazards model was used to estimate the association between having the residence in a lagging state and HF incidence and survival after HF diagnosis. For the HF incidence, the first diagnosis of HF was used as event, and age of diagnosis was used as time variable; for the survival after HF diagnosis, the death after HF diagnosis was used as event, and age of death was used as time variable. For both incidence and survival models, the residence in the lagging states was used as the predictor with the residence in the leading states as reference. Models for incidence and survival were analyzed for the total population and by sex- and race-specific subgroups stratified by age range (65–79 and 80+). In models for survival in the age 80+ group, we further stratified by age at HF diagnosis: < 80 and 80+. A sensitivity analysis was also conducted when including California and New York in the leading states (the results are shown in Supplemental eFigure 1). Results of Cox models for White and Black were included in the main results, and the results for other races (i.e., Hispanic, Asian, Native American, and others) were added in supplementary results (Supplemental eFigure 2). All analyses were performed using SAS software, version 9.4 (SAS Institute, Inc., Cary, NC).

## Results

Data from Medicare show that in the lagging states the proportion of males increased from 39.8% in 2000 to 44.5% in 2017 with the proportions of Whites and Blacks changing from 84.5% to 84.1% and from 12.0% to 11.5%, respectively. In the leading states, the proportion of males varied from 42.2% in 2000 to 45.6% in 2017, Whites from 88.7% to 85.7%, and Blacks from 3.6% to 4.4% (Supplemental eTable 1).

### Certificate-based mortality (CBM)

**Fig. 1:**
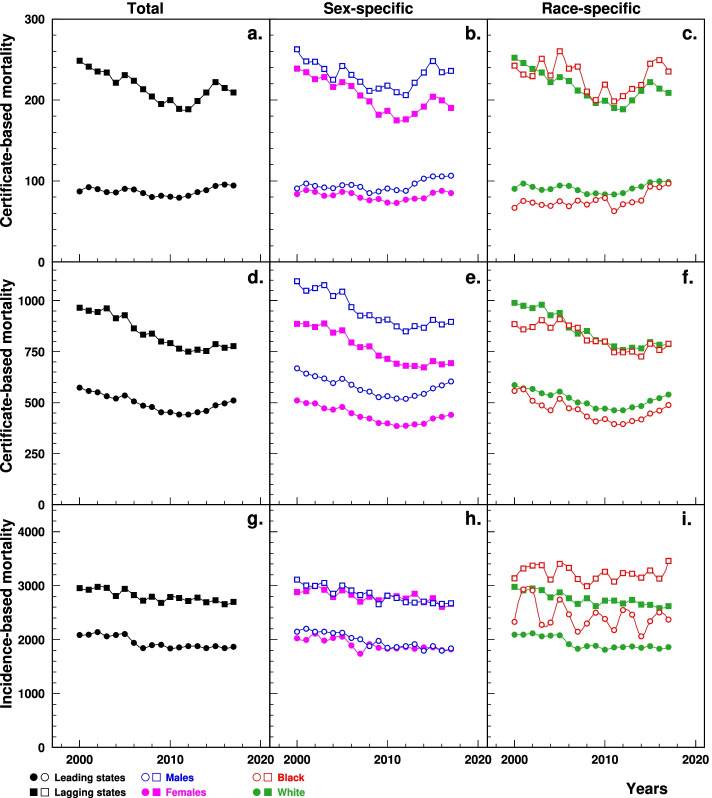
Temporal trend of age-standardized CBM-UCD (**a, b, c**), CBM-MCD (**d, e, f**) and IBM (**g, h, i**) of HF (per 100,000) among elderly aged 65+ in the leading and lagging U.S. states, 2000–2017, overall, by sex and race. Note: ^1^CBM-UCD=Death certificate-based mortality for underlying cause of death of HF, CBM-MCD = Death certificate-based mortality for multiple causes of death including HF, IBM = Incidence-based mortality, HF = heart failure. ^2^Data of the top six figures were derived from CDC WONDER (https://wonder.cdc.gov/); data for the bottom three figures were derived from 5% Medicare File of Service Use

Data from CDC WONDER show that the age-standardized HF CBM-UCD in the lagging states (per 100,000, from 248.6 in 2000 to 209.3 in 2017) was significantly higher than that in the leading states (86.9 to 94.2) (Fig. 1a). The age-standardized CBM-MCD in the lagging states declined from 965.9 in 2000 to 749.4 in 2012 followed by an increase to 777.2 in 2017. These changes were more pronounced than those in the leading states that declined from 574.1 in 2000 to 441.4 in 2011 and then increased to 511.3 in 2017 (Fig. 1d). The between-the-state difference decreased over time for both CBM-UCD and CBM-MCD. Similar patterns of CBM-UCD and CBM-MCD were shown by sex (Fig. 1b, e) and race (Fig. 1c, f), respectively.

### Incidence-based mortality (IBM)

Data from Medicare show that the age-standardized HF IBM in the lagging states was significantly higher (per 100,000, varying from 2953.4 in 2000 to 2697.7 in 2017) than in the leading states (2083.7 to 1866.5), with a plateau in recent decade (Fig. 1g). Similar patterns were observed in both sex groups (Fig. 1h) and in Whites with fluctuations in Blacks (Fig. 1i).

### HF incidence

**Fig. 2:**
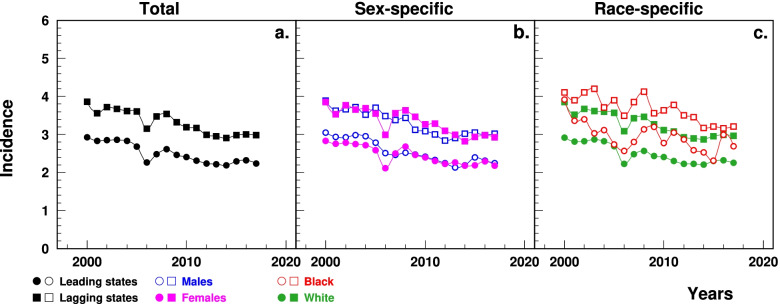
Temporal trend of age-standardized incidence of HF (%) among elderly aged 65+ in the leading and lagging U.S. states, 2000–2017, overall, by sex and race. Note: ^1^HF = heart failure. ^2^Data were derived from 5% Medicare File of Service Use. ^3^The sudden decline in 2005–2006 that particularly occurred among females was associated with the Medicare Policy change (https://www.liebertpub.com/doi/10.1089/jwh.2012.3777)

Medicare data show that the age-standardized HF incidence was significantly higher in the lagging states (3.9% in 2000 to 3.0% in 2017) than in the leading states (2.9 to 2.2%), with both states having an increase trend after 2014 (Fig. 2a). The between-the-state difference decreased after 2007. Sex- (Fig. 2b) and race-specific (Fig. 2c) patterns also demonstrated higher rates in the lagging states.

### HF prevalence

**Fig. 3:**
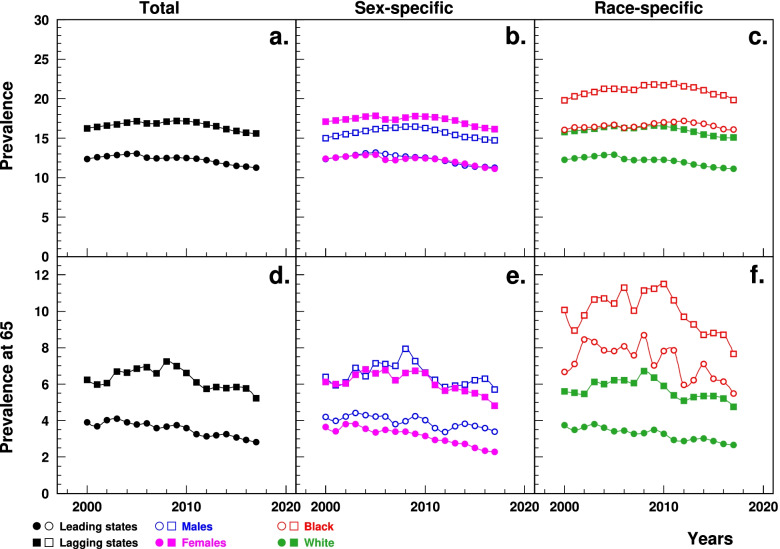
Temporal trend of age-standardized prevalence of HF among elderly aged 65+ (**a, b, c**) and prevalence of HF at age 65 (**d, e, f**) (%) in the leading and lagging  U.S. states, 2000–2017, overall, and by sex and race. Note: ^1^HF = heart failure. ^2^Data were derived from 5% Medicare File of Service Use. ^3^The sudden decline in prevalence in 2005–2006 that particularly occurred among females was associated with the Medicare Policy change (https://www.liebertpub.com/doi/10.1089/jwh.2012.3777)

The age-standardized HF prevalence estimated from Medicare data was significantly higher in the lagging states (ranging from 16.2% in 2000 to 15.6% in 2017) than the leading states (12.4 to 11.3%), with a recent gradual decline in both groups of the states (Fig. 3a). The age-standardized HF prevalence at age 65 was also significantly higher in the lagging states than the leading states (6.2% in 2000 to 5.2% in 2017 vs. 3.9 to 2.8%), with both states showing recent declines (Fig. 3d). The between-the-state difference in prevalence and prevalence at age 65 decreased after 2009 and 2008, respectively. Sex-(Fig. 3b, Fig. 3e) and race-specific (Fig. 3c, Fig. 3f) patterns also showed higher prevalence and prevalence at age 65 in the lagging states.

### Survival after HF diagnosis

**Fig. 4:**
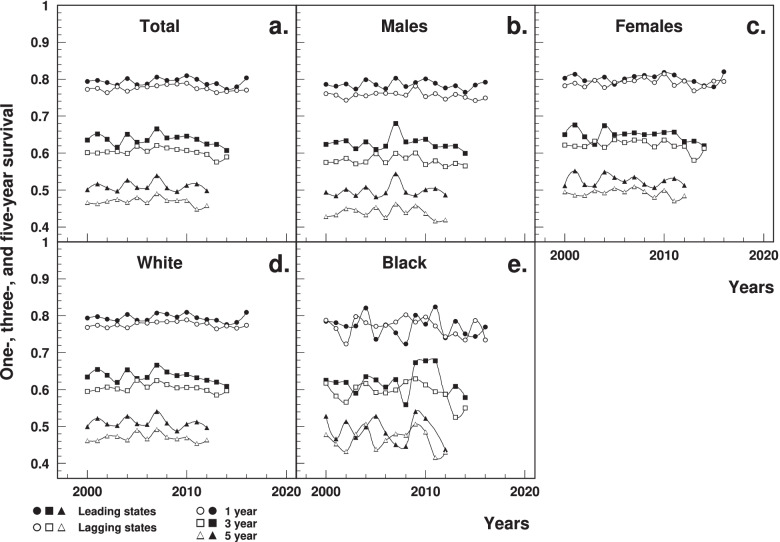
Temporal trend of age-standardized one-year, three-year and five-year survival rates after a HF diagnosis among elderly aged 65+ in the leading and lagging U.S. states, 2000–2017, overall, and by sex and race. Note: ^1^HF = heart failure. ^2^Data were derived from 5% Medicare File of Service Use

The age-standardized one-year survival estimated using Medicare data was significantly lower in the lagging states (77.3% in 2000 to 77.1% in 2016) than that in leading states (79.3 to 80.4%), with a gradual recent decline in both states (Fig. 4a). The age-standardized three-year and five-year survival were significantly lower in the lagging states, both declining since 2007 (Fig. 4a). Between-the-state differences and declining trend were also observed by sex- and race-specific groups (Fig. 4b-e).

### Effects of residence in lagging states

**Fig. 5:**
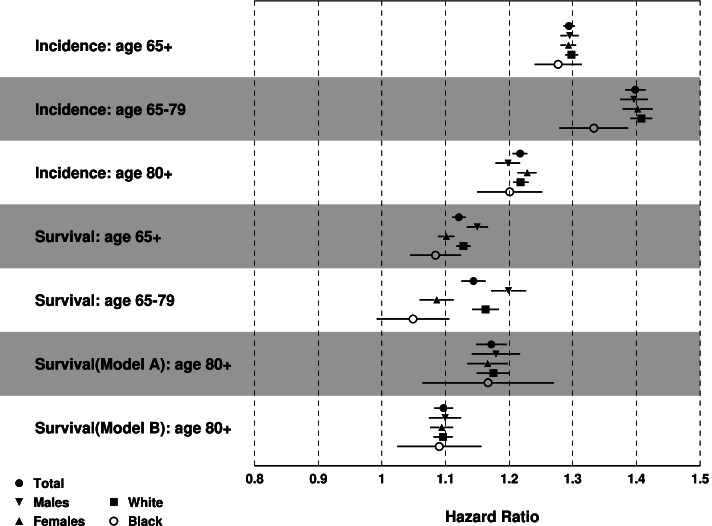
Results of multivariate Cox proportional hazards regression for HF incidence and survival after HF diagnosis: Adjusted hazards ratio (AHR) [95% CI] of  having residence in the lagging states. Note: ^1^Model A for age of diagnosis < 80 years old, Model B for age of diagnosis 80+. ^2^Age was controlled for incidence, and age of diagnosis was controlled for survival

Results of the Cox model showed that people aged 65+ living in lagging states had higher risk for HF incidence (Fig. 5): the Adjusted Hazards Ratio (AHR) [95% Confidence Interval (CI)]: 1.29 [1.29–1.30] for the total sample, 1.30 [1.28–1.31] for males, 1.29 [1.28–1.31] for females, 1.30 [1.29–1.31] for Whites, and 1.28 [1.24–1.32] for Blacks. Compared to people aged 65–79, patients aged 80+ showed less pronounced AHRs (1.22 [1.21–1.23] vs. 1.40 [1.38–1.41] for the total population). The lowest risk was observed in the age 80+ male stratum (1.20 [1.18–1.22]) and the highest risk was observed in the age 67–79 White stratum (1.41 [1.39–1.43]).

Individuals aged 65+ in the lagging states had higher risk of death after HF diagnosis: 1.12 [1.11–1.13] for total population, 1.15 [1.13–1.17] for males, and 1.10 [1.09–1.11] for females, 1.13 [1.12–1.14] for Whites, and 1.08 [1.05–1.13] for Blacks. The highest and lowest risks associated with residence in a lagging state were 1.20 [1.17–1.23] for males aged 65–79 years and 1.05 [0.99–1.11] for Black population aged 65–79.

The sensitivity analysis when including California and New York showed that compared to the leading states the populations of the lagging states had a higher risk of HF incidence as well as higher risk of death after HF diagnosis. More details could be found in Supplemental eFigure 1.

Results of Cox regression for other races were consistent with the total sample excepting Hispanic incidence (0.88 [0.77–1.00]) (Supplemental eFigure 2).

## Discussion

Our study found substantial geographic disparities in HF outcomes across the US: older adults aged 65+ in the lagging states had higher HF mortality, incidence, prevalence, and lower survival, with most of the disparities in mortality originating from differences in HF incidence, pre-existing prevalence of HF at age 65, and survival after HF diagnosis, accompanied with increasing incidence as well as declining prevalence and survival in HF patients in both leading and lagging states. The findings in this study are consistent with previous works on HF mortality [8, 9, 18] and incidence [19].

Our study has a significant advantage: it is based on the analysis of over 5 million Medicare beneficiaries which provides sufficient power for the analysis of relatively small geographic regions and is nationally representative sample of older U.S. adults aged 65+, covering all geographic regions in the U.S. and allowing for evaluation of both morbidity and mortality. Furthermore, this Medicare-based analysis is combined with death certificate data from CDC-WONDER to reduce the impact of the limitations associated with administrative data. Previous studies on geographic patterns of HF mortality were based on death certificate data only and did not investigate the associated epidemiological measures (e.g., incidence, prevalence) [8, 9]. Furthermore, most such studies were community-based cohort studies [13, 20] with poor generalizability to the total U.S. population. The single existing Medicare-based study [19] identified by the authors investigated the geographic disparities of HF in four U.S. census regions (i.e., Midwest, Northeast, South, and West) and only examined HF prevalence and incidence.

Our study showed that all hypothesized scenarios (higher incidence, poorer survival and higher prevalence at age 65) contributed to the geographic differences in HF mortality. Study results showed substantially higher pre-existing prevalence of HF in lagging states prior to Medicare eligibility. This phenomenon could be explained by the earlier onset of HF in the lagging states. Data from the Behavioral Risk Factor Surveillance System showed that the prevalence of coronary heart disease and myocardial infarction at ages younger than 65 was higher in the lagging states [21], which suggest a higher incidence and prevalence of HF before age 65 in the lagging states. The gap of prevalence at age 65 narrowed after 2008, which explained part of the observed decline in the gap in HF mortality. Early primary prevention efforts targeting HF risk factors in young and middle-aged adults in the lagging states are desirable.

Disparities of HF incidence - another important contributor to the geographic disparity - are often attributed to the differences in the distribution of associated risk factors (e.g., hypertension, diabetes) [19, 22], and their impacts [23]. We found that the incidence gap between the leading and lagging states was decreasing over the time period available for our study. However, this did not represent a beneficial trend as it was caused by relatively quicker incidence growth in the leading states rather than incidence reduction in the lagging states. This suggests the need for intensive prevention and awareness programs even in those states with relatively good epidemiological profiles.

Finally, HF survival was also lower in the lagging states, likely due to between-the-state differences in stage at diagnosis, access to/quality of healthcare, behavioral habits, and the prevailing comorbidity profiles [8–10, 18]. For example, data from the CDC showed that patients in lagging states had greater nonadherence to arterial hypertension treatment and cholesterol-lowering medication intake, as well as higher eligibility for cardiac rehabilitation coupled with low participation rates [24]. This suggests that tertiary prevention work targeting the treatment and management after HF onset is needed to improve the survival in the lagging states.

In addition to the substantial geographic disparities, we observed that the CBMs showed recent increases while the IBM sustained relatively stable, that may be associated with the administrative nature of Medicare data, where patients are followed up till the end of their enrollment instead of their deaths. Study results showed a decade-long plateau stage in HF IBM and decline in prevalence in both the leading and lagging states that may be attributable to joined effects from the increases in incidence and declines in prevalence at age 65 and survival. The recent increase in incidence may be attributed to the adoption of better testing methods [25], the increasing rates of obesity and diabetes [10], and some negative lifestyle changes (e.g., low physical activity) [4, 26]. The decline in prevalence at age 65 may be related to the declines in survival while the survival decline may be related to increasing levels of multimorbidity in the elderly as well as the increasing proportion of HF with preserved ejection fraction (HFpEF), a common subtype of HF among older patients that does not have a specific treatment [4, 26–29]. Although HFpEF is not ascertainable in our data, the overall trends in mortality are supportive of these findings.

Another explanation of the declining survival is the Hospital Readmissions Reduction Program (HRRP) that was discussed in 2007–2009, announced in 2010 and implemented in 2012. The HRRP aims to encourage hospitals to improve the quality of health care by imposing Medicare payment penalties on hospitals with higher-than-expected readmission rate, with three diseases initially covered, including HF, acute myocardial infarction, and pneumonia [30]. The penalties may lead the hospitals to take inappropriate care strategies, such as delaying patients’ readmission beyond day 30, increasing observation stays without admission, shifting inpatient care to outpatient/emergency care [31], increasing the coding disease severity [32], that may adversely affect the health outcomes in HF patients [31]. Previous studies based on the data from the Medicare Beneficiaries showed that the 30-day and 1-year HF mortality rates were higher after the implementation of the HRRP [31, 33, 34], despite the successful reduction of the hospital readmission rates [31, 33, 35, 36]. Further studies are needed to investigate this issue.

The study results showed greater geographic disparities in HF outcomes among Whites than Blacks, which might be related to the differences in risk factor distribution [37] and the impacts of these factors [38]. On the contrary, Hispanics in the leading states had higher incidence, that can be partially explained by associated higher prevalence of risk factors in the leading states than the lagging states [37, 39] as well as lower access to health care, lower coverage of health insurance [40] and hospitalization rate [41] in the lagging states, that may lead to an underdiagnosis of HF. More data are needed to investigate the causes.

In our study, patients aged 80+ had less pronounced between-the-state differences in HF incidence compared to patients aged 65–79 years old. Possible explanations could be that more people with HF risk factors do not survive to age 80 in the lagging states, that may lead to a smaller between-the-state differences for the prevalence of obesity, diabetes, and arterial hypertension among patients aged 80+ than patients aged 65–79 years old [21] and respective age-specific impacts of these factors on HF incidence [42].

This study has an important limitation: information on specific subtypes of HF is limited in Medicare claims. This reduces the generalizability of our findings to patient groups with well-defined HF subtypes and suggests the need for more granular studies.

Based on the results of this study, future studies will apply trend decomposition analyses (such as partitioning [43–46]) to verify the causes of these trends and the relative changes in the magnitude of their effects over time as well as the role of complementary trends in related comorbid conditions (e.g., diabetes, coronary heart disease, and myocardial infarction). Furthermore, the quantification of differences in treatment and medication prescription/utilization patterns (which can be derived from Medicare Part D data) can provide further insight into the mechanisms generating these disparities.

## Conclusion

This study quantified substantial geographic disparities in HF outcomes across the US with lagging states having significantly higher mortality, incidence, prevalence, and lower survival among adults aged 65+. Geographic disparities in HF mortality may be explained in a great extent through geographic patterns of HF incidence, its prevalence among Medicare enrollees, and survival of patients with HF. That allows for suggestion of optimization of modifiable risk factors that increase the risk of HF, the efforts to improve timely ascertainment of the condition, and better treatment and management after diagnosis.

## Supplementary Information

**Additional file 1: eTable 1.** Characteristics of the study sample, n (%). **eFigure 1.** Results of multivariate Cox proportional hazards regression for HF incidence and survival after HF diagnosis: Adjusted hazards ratio (AHR) [95% CI] of residence in the lagging states when New York and California were included in the leading states. **eFigure 2.** Results of multivariate Cox proportional hazards regression for HF incidence (upper panel) and survival after HF diagnosis (lower panel) among Hispanics, Asian, Native Americans and other races: Adjusted hazards ratio (AHR) [95% CI] of residence in the lagging states.

## Data Availability

The data that support the findings of this study are available from Centers for Medicare & Medicaid Services, but restrictions apply to the availability of these data which were used under license for the current study and so are not publicly available. Data are however available from the authors upon reasonable request and with permission of Centers for Medicare & Medicaid Services.
