# Supplementary material for: Epidemiology of geographic disparities in heart failure among US older adults: a Medicare-based analysis
Source: BMC Public Health. 2022 Jul 1;22:1280. doi: 10.1186/s12889-022-13639-2 (PMC9248157; doi:10.1186/s12889-022-13639-2)
Supplement: Supplementary file 1 — Additional file 1: eTable 1. Characteristics of the study sample, n (%). eFigure 1. Results of multivariate Cox proportional hazards regression for HF incidence and survival after HF diagnosis: Adjusted hazards ratio (AHR) [95% CI] of residence in the lagging states when New York and California were included in the leading states. eFigure 2. Results of multivariate Cox proportional hazards regression for HF incidence (upper panel) and survival after HF diagnosis (lower panel) among Hispanics, Asian, Native Americans and other races: Adjusted hazards ratio (AHR) [95% CI] of residence in the lagging states. [file 12889_2022_13639_MOESM1_ESM.docx]

**Supplement**

**eTable 1. Characteristics of the study sample, n (%)**

| **Variable** | **2000** | **2002** | **2004** | **2006** | **2008** | **2010** | **2012** | **2014** | **2016** | **2017** |
| --- | --- | --- | --- | --- | --- | --- | --- | --- | --- | --- |
| ***Leading states*** | | | | | | | | | | |
| **Sex** |  |  |  |  |  |  |  |  |  |  |
| Male | 92376  (42.17) | 97219 (42.58) | 100486 (42.91) | 104308 (39.58) | 104888 (40.56) | 107498 (41.33) | 111571 (42.31) | 116829 (42.92) | 117300 (44.94) | 120204 (45.55) |
| Female | 126702 (57.83) | 131104 (57.42) | 133684 (57.09) | 159196 (60.42) | 153730 (59.44) | 152625 (58.67) | 152104 (57.69) | 155404 (57.08) | 143688 (55.06) | 143678 (54.45) |
| **Race** |  |  |  |  |  |  |  |  |  |  |
| White | 194322 (88.70) | 202072 (88.50) | 206738 (88.29) | 232716 (88.32) | 228164 (88.22) | 228483 (87.84) | 230532 (87.43) | 235936 (86.67) | 224366 (85.97) | 226046 (85.66) |
| Black | 7869 (3.59) | 8644 (3.79) | 9265 (3.96) | 10612 (4.03) | 10429 (4.03) | 10904 (4.19) | 11072 (4.20) | 11691 (4.29) | 11302 (4.33) | 11487 (4.35) |
| Hispanic | 5052 (2.31) | 5271 (2.31) | 5386 (2.30) | 6109 (2.32) | 6007 (2.32) | 6259 (2.41) | 6206 (2.35) | 6341 (2.33) | 5782 (2.22) | 5634 (2.14) |
| Asian | 3479 (1.59) | 3641 (1.59) | 3724 (1.59) | 4198 (1.59) | 4179 (1.62) | 4217 (1.62) | 4303 (1.63) | 4557 (1.67) | 4569 (1.75) | 4695 (1.78) |
| Native American | 817 (0.37) | 917 (0.40) | 970 (0.41) | 1112 (0.42) | 1137 (0.44) | 1228 (0.47) | 1325 (0.50) | 1467 (0.54) | 1549 (0.59) | 1609 (0.61) |
| Others | 7089 (3.24) | 7397 (3.24) | 7772 (3.32) | 8446 (3.21) | 8405 (3.25) | 8333 (3.20) | 8267 (3.14) | 8405 (3.09) | 7962 (3.05) | 8147 (3.09) |
| Unknown | 450 (0.21) | 381 (0.17) | 315 (0.13) | 311 (0.12) | 297 (0.11) | 699 (0.27) | 1970 (0.75) | 3836 (1.41) | 5458 (2.09) | 6264 (2.37) |
| ***Lagging states*** | | | | | | | | | | |
| **Sex** |  |  |  |  |  |  |  |  |  |  |
| Male | 73956 (39.79) | 75527 (40.16) | 77291 (40.73) | 79965 (37.71) | 78777 (38.73) | 79806 (39.49) | 83078 (40.61) | 86251 (41.55) | 87230 (43.78) | 89601 (44.47) |
| Female | 111923 (60.21) | 112550 (59.84) | 112465 (59.27) | 132105 (62.29) | 124617 (61.27) | 122262 (60.51) | 121485 (59.39) | 121353 (58.45) | 111995 (56.22) | 111871 (55.53) |
| **Race** |  |  |  |  |  |  |  |  |  |  |
| White | 156995 (84.46) | 159228 (84.66) | 160874 (84.78) | 180568 (85.15) | 173736 (85.42) | 172310 (85.27) | 174112 (85.11) | 175513 (84.54) | 167957 (84.31) | 169377 (84.07) |
| Black | 22223 (11.96) | 22028 (11.71) | 21905 (11.54) | 23981 (11.31) | 22428 (11.03) | 22507 (11.14) | 22823 (11.16) | 23693 (11.41) | 22619 (11.35) | 23148 (11.49) |
| Hispanic | 306 (0.16) | 343 (0.18) | 359 (0.19) | 415 (0.20) | 434 (0.21) | 477 (0.24) | 512 (0.25) | 602 (0.29) | 695 (0.35) | 733 (0.36) |
| Asian | 437 (0.24) | 497 (0.26) | 555 (0.29) | 673 (0.32) | 702 (0.35) | 789 (0.39) | 841 (0.41) | 944 (0.45) | 984 (0.49) | 1029 (0.51) |
| Native American | 1345  (0.72) | 1532  (0.81) | 1616  (0.85) | 1925  (0.91) | 1910  (0.94) | 1923  (0.95) | 2011  (0.98) | 2129  (1.03) | 2089  (1.05) | 2120  (1.05) |
| Others | 4148  (2.23) | 4121  (2.19) | 4180  (2.20) | 4242  (2.00) | 3972  (1.95) | 3730  (1.85) | 3519  (1.72) | 3305  (1.59) | 2985  (1.50) | 2892  (1.44) |
| Unknown | 425  (0.23) | 328  (0.17) | 267  (0.14) | 266  (0.13) | 212  (0.10) | 332  (0.16) | 745  (0.36) | 1418  (0.68) | 1896  (0.95) | 2173  (1.08) |

Note: Data in the table were derived from the 5% Medicare claims data.

**eFigure 1. Results of multivariate Cox proportional hazards regression for HF incidence and survival after HF diagnosis: Adjusted hazards ratio (AHR) [95% CI] of residence in the lagging states when New York and California were included in the leading states**

**eFigure 2. Results of multivariate Cox proportional hazards regression for HF incidence (upper panel) and survival after HF diagnosis (lower panel) among Hispanics, Asian, Native Americans and other races: Adjusted hazards ratio (AHR) [95% CI] of residence in the lagging states**

Note: ^1^HF=Heart failure. ^2^Data were derived from 5% Medicare File of Service Use. ^3^Age was controlled for incidence, and age of diagnosis was controlled for survival.

**Supplementary Methods:**

Please refer to the following materials:

(1) The supplementary methods of the publication: Yu B, Akushevich I, Yashkin A, Kravchenko J. Epidemiology of geographic disparities of myocardial infarction among older adults in the United States: Analysis of 2000-2017 Medicare data. *Frontiers in Cardiovascular Medicine*. 2021, https://doi.org/10.3389/fcvm.2021.707102.

(2) Akushevich I, Kravchenko J, Ukraintseva S, Arbeev K, Yashin AI. Age Patterns of Incidence of Geriatric Disease in the US Elderly Population: Medicare‐Based Analysis. *Journal of the American Geriatrics Society.* 2012;60(2):323-327.
